# Systematic nurse-led consultations based on electronic patient-reported outcomes for women with endometrial or ovarian cancer during chemotherapy—a feasibility study

**DOI:** 10.1007/s00520-025-09875-y

**Published:** 2025-09-17

**Authors:** Mille Guldager Christiansen, Helle Pappot, Mary Jarden, Anders Tolver, Hjalte Søberg Mikkelsen, Mansoor Raza Mirza, Karin Piil

**Affiliations:** 1 Department of Oncology, Centre for Cancer and Organ Diseases, Copenhagen University Hospital, Rigshospitalet, Copenhagen, Denmark; 2 Department of Clinical Medicine, Faculty of Health and Medical Sciences, University of Copenhagen, 2200 Copenhagen, Denmark; 3 Department of Haematology, Centre for Cancer and Organ Diseases, Copenhagen University Hospital, Rigshospitalet, Copenhagen Denmark; 4 Data Science Laboratory, Department of Mathematical Sciences, University of Copenhagen, Copenhagen, 2100 Denmark; 5 School of Nursing, Faculty of Health Sciences, Curtin University, Perth, Western Australia, Australia

**Keywords:** Gynecological cancer, Symptom assessment, Electronic monitoring, Nurse-led consultations, Electronic patient-reported outcomes

## Abstract

**Purpose:**

This study aimed to investigate the feasibility of nurse-led consultations based on ePRO for women with endometrial or ovarian cancer receiving chemotherapy.

**Methods:**

This was a prospective single-cohort feasibility study. The patients responded to weekly ePRO using the Elekta Kaiku platform, which was adapted to Danish. At selected time points during chemotherapy, nurses conducted nurse-led consultations replacing physicians. The primary outcome was the patient completion rate of ePRO reporting in the Elekta Kaiku platform. Secondary outcomes included assessing safety, acceptability, practicability, usability, patient satisfaction with ePRO, and patient symptom burden.

**Results:**

Twenty patients were included, with a mean age of 59 years. A total of 320 ePRO reports were received, equivalent to an ePRO response of 18 times per patient during six cycles of chemotherapy. The results showed a high weekly completion rate (87%), high patient satisfaction with ePRO, and a continuous high symptom burden. Nurse-led consultations achieved a success rate of 55% in adhering to scheduled appointments. The primary contributing factors to this issue were the inadequate internal workflows and the complex symptom burden experienced by the patients.

**Conclusion:**

This study indicates that weekly ePRO reporting in conjunction with nurse-led consultations may be feasible in this population. The use of ePRO revealed a high level of symptoms reported by patients throughout the treatment cycles. The results emphasize the importance of clinicians in proactively and systematically intervening at an early stage to prevent symptom escalation. However, the small sample size limits the generalizability of this study.

**Trial registration:**

This study was registered at the Capital Region of Denmark (P-2021–179) and approved the 10/03/2021. ClinicalTtrials.gov ID: NTCC04945187.

**Supplementary Information:**

The online version contains supplementary material available at 10.1007/s00520-025-09875-y.

## Introduction

Approximately 8% of all new cases of cancer in women worldwide are endometrial and ovarian cancers [1]. The treatment is determined by the stage of the disease and consists of a combination of extensive surgery and chemotherapy. First-line chemotherapy for ovarian cancer includes platinum-based chemotherapy (carboplatin and paclitaxel/docetaxel) every 3 weeks, either as adjuvant or neoadjuvant treatment. Further, for high-risk endometrial cancer, adjuvant chemotherapy (paclitaxel and carboplatin) is recommended [2]. Throughout the course of treatment, the women may experience physical symptoms including peripheral neuropathy, fatigue, bloating, and constipation [3]. Additionally, psychological symptoms such as anxiety, cognitive dysfunction, and depression are common [4]. As more treatment is provided in outpatient settings, clinicians have less time to assess the severity of symptoms and initiate supportive care [5]. Further, healthcare professionals often underestimate patients’ symptomatic adverse events, resulting in undetected symptoms [6].

Optimal symptom assessment and evidence-based management require the skills of a multidisciplinary team. Oncological specialized nurses, specifically those educated and experienced in providing self-management support, play a crucial role in reducing physical and emotional symptom severity and improving the quality of life for patients [7]. Therefore, specialized cancer nurses are in a prominent position with their knowledge and experience to address and facilitate symptom management and support patient empowerment [8]. Nurses may have the potential to substitute for doctors at certain stages of treatment, and this type of consultation has been successfully implemented in other cancer settings, resulting in high levels of patient satisfaction [9].

Patient-reported outcomes (PROs) encourage patients to report their health status directly without clinicians’ interpretation [10]. An advantage of PROs is the early identification of symptoms, enabling prompt intervention and preventing symptoms from intensifying [11]. Thus, the use of PROs may improve patient-clinician communication, patient satisfaction, and symptom management [6]. Electronic PROs (ePROs) can enhance the quality of care and enable clinicians to better identify unmet symptom needs and trends over time, guiding optimal symptom interventions [12]. Further, when combined with self-management guidance and clinical algorithms for management, ePRO may improve physical well-being and self-efficacy [13]. The evidence regarding ePRO in gynecological cancer is limited; however, in the eRAPID study [13], women with a variety of gynecological diseases undergoing chemotherapy were enrolled [13], and ePRO was further examined in the follow-up phase [14, 15] and has been proven feasible and acceptable [14, 15].

However, to the best of our knowledge, the combination of ePRO and nurse-led consultations during chemotherapy in this population has not yet been investigated. Therefore, this study aimed to investigate the feasibility of nurse-led consultations based on ePRO among women undergoing platinum-based chemotherapy for ovarian or endometrial cancer.

## Materials and methods

This study is part of a larger study, and the original protocol has been published previously [16]. This manuscript adheres to the CONSORT 2010 statement: extension to randomized pilot and feasibility trials checklist [17] (Supplementary File 1).

### Study design

This was a single-site prospective feasibility study in terms of testing nurse-led consultations based on ePRO. This study was carried out at the Department of Oncology, Copenhagen University Hospital, Denmark.

### Outcomes

#### Primary outcome

The primary outcome was the patients’ completion rate of ePRO reporting per week. This was defined as the proportion of patients who completed ePRO self-reporting at each weekly time point divided by the number of ePROs sent to the patients [18]. If patients completed multiple ePRO responses per week, only one response per week was included in the analysis to maintain consistency.

#### Secondary outcome

Feasibility was evaluated based on general and study-specific parameters [19, 20]: including safety (timely management of moderate or severe symptoms, nurses consulting a physician in case of doubt, and ensuring patient data protection), acceptability (adherence, recruitment, conduction of nurse-led consultations and attrition), practicability (telephone contacts and documentation), usability and patient satisfaction, and patients’ symptom burden. Safety was assessed by the number of unexpected events related to ePRO reporting and nurse-led consultations. The adherence rate was the proportion of participants replying to ≥ 80% of the weekly ePRO [18]. The recruitment rate was defined as the proportion of informed patients giving consent by dividing the number of potential participants. A recruitment rate of > 50% was considered sufficient. The attrition rate was defined as the proportion of patients who dropped out of the intervention, leaving no outcome data available. To investigate the satisfaction and patient acceptance of ePRO, the Patient Feedback Form was used at the end of treatment (6 months) [21].

### Participants and recruitment

The recruitment period was from July 2022 to January 2023. The study included women aged ≥ 18 with a diagnosis of endometrial or ovarian cancer, planned to receive chemotherapy (docetaxel/paclitaxel, carboplatin, ± bevacizumab), speaking and understanding Danish, and having a valid email address and a digital device [16]. Patients with severe cognitive or mental illness were excluded. Clinicians initially approached all eligible patients, and if they expressed additional interest, they were given further information by the primary investigator. All patients were provided with written information about the study. The inclusion continued consecutively until (*n* = 20) patients were recruited. The sample size estimation was based on recommendations for pilot and feasibility studies [19, 20].

### Intervention

The intervention consisted of nurse-led consultations based on ePRO. The patients reported symptoms weekly in a designated ePRO platform, Elekta Kaiku Ltd. [22], and the ePRO-based nurse-led consultations replaced physician-led consultations on pre-selected time points.

The platform included a symptom questionnaire containing 21 symptoms selected from the PRO-CTCAE library [23, 24]. The 21 symptoms were classified as physical, psychological, or cognitive (Supplementary File 2) [23]. These were selected following a two-phase sequential multi-method study comprising literature reviews, a national expert panel, focus group interviews with physicians and specialized oncology nurses, and consultations with a patient advisory board [23].

A threshold algorithm for symptoms was developed, inspired by Tolstrup et al. [25] and colored according to severity: green (mild symptoms), orange (moderate symptoms), red (severe symptoms). The algorithm guided the responses to the patients, including self-management guidance to empower the patients to manage symptoms at home. Before commencing the study, the Elekta Kaiku ePRO platform was customized to accommodate the Danish context. Patients could use the ePRO platform remotely, choosing between a mobile application or a web browser, according to their preferences [22]. Patients had unlimited access to the Elekta Kaiku ePRO platform and could report symptoms as often as they wished, but at a minimum once a week. They also had the opportunity to add additional symptoms. Patients received weekly reminders powered by the Elekta Kaiku ePRO platform [22].

**Fig. 1:**
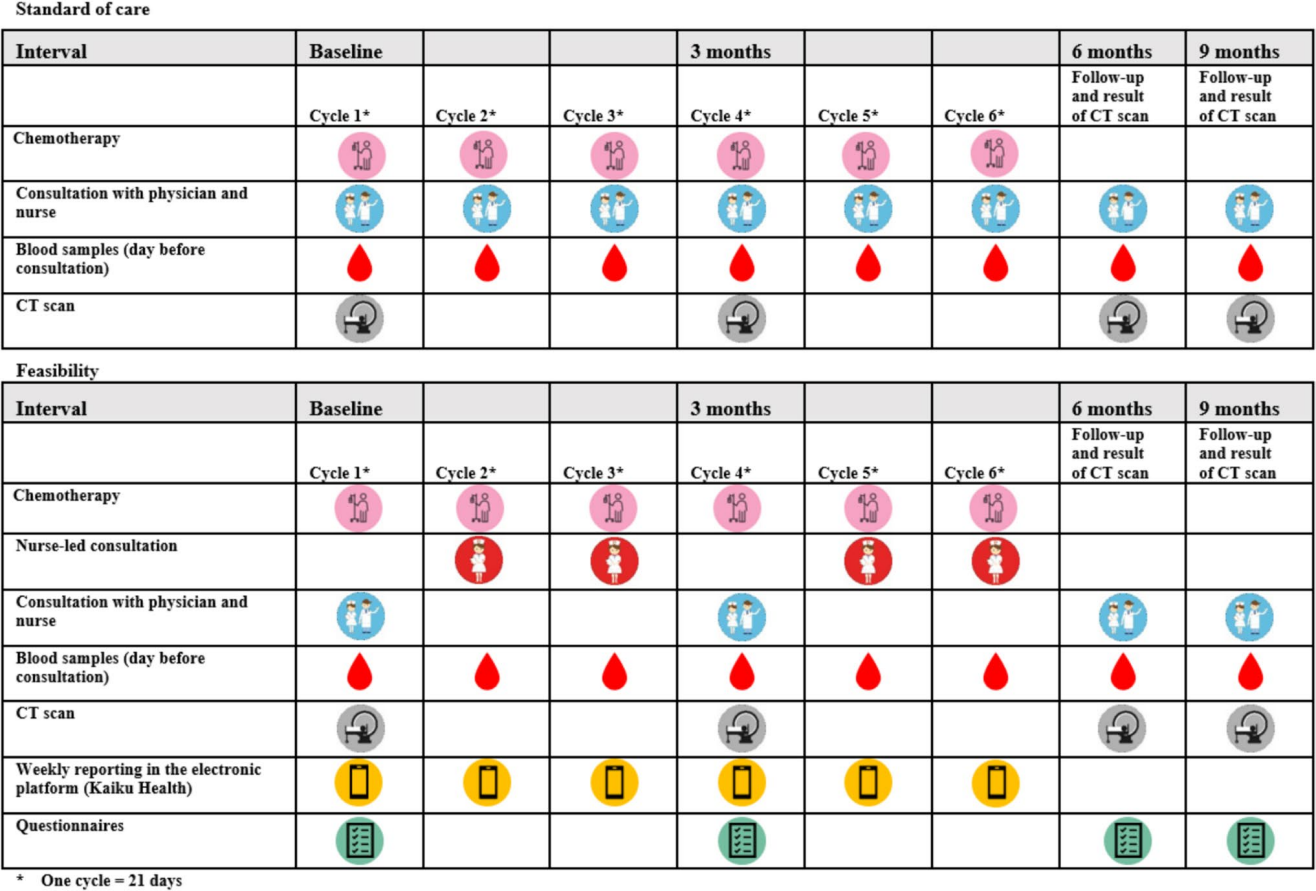
The difference between the standard of care and the feasibility study

The nurse-led consultations took place at two to four designated time points, as outlined in Fig. 1, depending on the treatment trajectory (neoadjuvant or adjuvant). They included assessment of adverse events (AE) based on Common Terminology Criteria for Adverse Events (CTCAE) [26], evaluation of blood samples, and providing the patient with self-management guidance and support. Physicians and nurses had access to a printout of the patient’s ePRO responses for each scheduled hospital visit. A coordinating nurse reviewed the patients’ ePRO reports (Monday to Friday). The nurses were notified by email generated by the ePRO Elekta Kaiku ePRO platform if the patient’s ePRO response exceeded the predetermined threshold. Patients who presented severe or concerning symptoms were advised to notify the Department of Oncology promptly. In the event that they did not, the nurses initiated contact with them in accordance with a predetermined study-specific algorithm inspired by others [25].

Before study initiation, several initiatives were planned to improve clinician adherence such as an introduction to the ePRO platform, a training program for the specialized nurses conducting the consultations, staff meetings, guidelines and instructions describing study details, and unlimited support from the principal investigator.

### Data collection

Demographic data (e.g., age, disease) were collected at baseline either electronically via the web-based platform REDCap [27] or on paper, depending on the patient’s preference.

Adverse events (AE) were assessed according to the standard procedure following Common Terminology Criteria for Adverse Events (CTCAE) prior to each cycle of chemotherapy [26]. Clinical data related to diagnosis, treatment, and stage of disease were extracted from the electronic medical report. Further, the electronic medical reports also extracted study-specific data, including the number of nurse-led consultations and telephone contacts. The clinical data were stored in REDCap [27]. A conditional branching logic was employed for the 21 PRO-CTCAE symptoms with two or more attributes (severity, interference, or frequency) [23, 28]. Usage data of the patient’s ePRO reporting were extracted from Elekta Kaiku [22]. Further, as described in the protocol [16], we collected multiple PROs at baseline, 3-, 6-, and 9-month intervals; however, these data are not included in this study.

### Statistical analyses

The demographic and clinical characteristics were presented using descriptive statistics. The categorical variables were summarized using frequency and percentages, while the numerical data were described using the mean and range.

Basch et al. calculated a composite score for each PRO-CTCAE symptom by considering all attributes, with values ranging from 0 to 3 [28, 29]. The composite score classifies symptoms into four categories: none (0), mild (1), moderate (2), and severe (3). Higher composite scores indicate more severe symptoms [28]. In addition, the proportion of patients with PRO-CTCAE symptoms of grade 2 or above was calculated for each week. The statistical analysis was carried out using R.

### Ethical considerations

The study was conducted following the General Data Protection Regulation and Helsinki Declaration [30]. Approval was granted by the Danish Data Protection Agency (file no.: P-2021–179) and was registered at ClinicalTrials.gov (NCT04945187). All study participants provided written informed consent following the Helsinki Declaration [30].

## Results

In the period from June 2022 to January 2023, *n* = 47 consecutive patients were assessed for eligibility, *n* = 10 patients were excluded (not being Danish speaking, having no email, or cognitive deficit), and *n* = 16 patients declined participation due to feeling too overwhelmed. In total, 21 patients were included, but one did not complete the baseline questionnaire and withdrew. Hence, a total of 20 patients were included, resulting in a recruitment rate of (20/37) = 54%. The participants´ characteristics are shown in Table 1.

**Table 1:** The participants’ characteristics (*n* = 20)

	*n* = 20
Age	
Mean in years(range)	59 (33–77)
Marital status, ***n*** (%)	
Married	15 (75)
Living alone	5 (25)
Highest level of education, *n* (%)	
Skilled worker	6 (30)
University undergraduate	6 (30)
University graduate	8 (40)
Employment, *n* (%)	
Full-time work	8 (40)
Part-time work	1 (5)
Retired/unemployed sick leave	11 (55)
Digital literacy, *n* (%)	
Unable to respond to emails or read digital mail from the public sector or private companies	1 (5)
Able to respond to emails or read digital posts from the public sector and private companies	7 (35)
Manage technology very safe/expert	12 (60)
Origin of cancer, *n* (%)	
Ovarian cancer	17 (85)
Endometrial cancer	3 (15)
Diagnostic stage according to FIGO, *n* (%)	
Stage I–II	5 (25)
Stage III–IV	14 (70)
Unknown	1 (5)
Number of chemotherapy cycles, *n* (%)	
3–4 cycles	4 (20)
5–6 cycles	16 (80)
Primary chemotherapy regimen, *n* (%)	
Docetaxel/carboplatin	12 (50)
Paclitaxel/carboplatin	8 (40)

*FIGO* International Federation of Gynecology and Obstetrics

The patients had a mean age of 59 and were generally proficient internet and technology users. The majority (70%) were diagnosed with ovarian cancer. Five patients with ovarian cancer were also included in a surgical clinical trial and received hyperthermic intraperitoneal chemotherapy (OVHIPEC-2) in combination with surgery [31]. A patient advisory board, comprising five women with a history of either ovarian or endometrial cancer, actively contributed to the development of the Danish adaptation of the Danish Elekta Kaiku platform [22].

### Primary outcome

#### Completion rate

**Fig. 2:**
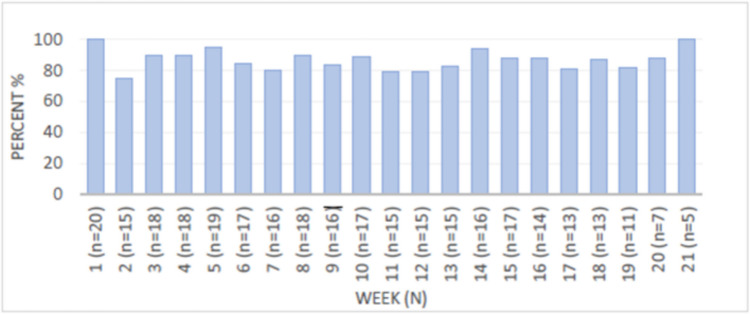
The weekly completion rate of ePRO reporting

In total, 365 weekly ePRO questionnaires were sent to the patients between 9 and 21 times per patient, and 320 ePRO questionnaires were completed in Elekta Kaiku, resulting in an ePRO completion rate of 87% (Fig. 2).

### Secondary outcomes

#### Feasibility

During the study period, no safety issues were identified.

#### Acceptability

The adherence rate of patients responding to ≥ 80% of the weekly ePRO during the period was 86%, and the mean number of completed ePROs was 18 (range 4–40) ePRO reports per patient. The recruitment rate was concluded within 6 months, indicating a willingness and interest among participants to engage in this study. The attrition rate was 4.7%, with only one patient withdrawing due to feeling overwhelmed.

Six patients used the opportunity to provide additional information by writing in a free-text box, describing their symptoms between 1 to 12 times. The additional symptoms reported were hives, fever and infection, nail rupture, cystitis, taste changes, nosebleeds, dizziness, abdominal pain, and other information to the nurses.

A total of 66 consultations were scheduled as potential nurse-led consultations replacing the physician. After a mid-way evaluation after 12 weeks of recruitment, we reduced the number of nurse-led consultations due to the complexity of the patients, as specified in Table 2. Therefore, 36 consultations were conducted by nurses as planned, representing a success rate of (36/66) = 55%, as specified in Table 2.

**Table 2:** Overview of the scheduled and conducted nurse-led consultations

	The scheduled number of nurse-led consultations for the specific treatment trajectory	Numbers of patients	Total scheduled nurse-led consultations	Total number of nurse-led consultations conducted as scheduled
Study initiation				
Adjuvant setting	4	9	36	21
Neo-adjuvant setting	3	3	9	5
After adjustment				
Adjuvant setting	3	5	15	9
Neo-adjuvant setting	2	3	6	1
		**Total**	**66**	**36**

Four patients did not attend nurse-led consultations due to the complexity of their symptoms, and 16 patients had between one and four nurse-led consultations. Medical conditions necessitating the intervention of a physician, such as the prescription of preventive drugs to decrease immunological hypersensitivity, infections, a high number of symptoms, the need to adjust medication dosage due to peripheral neuropathy, and genetic testing, hindered the potential for nurse-led consultations.

One patient contacted the principal investigator regarding a technical problem that arose with the mobile phone application. The issue was rectified by replacing the older device that was the source of the problem.

### Practicability

The Department of Oncology did not receive any extra nursing resources for this study; thus, the ePRO was incorporated into the existing workflow. The patients had a mean of seven telephone contacts (range 2–13) with the nurses at the Department of Oncology throughout their cancer treatment trajectory, from the beginning of treatment until after the last cycle. Further, the mean number of phone calls linked to reporting in the ePRO platform was 4 (range 2–9).

### Usability and patient acceptance/satisfaction with ePRO

Patients, on mean, accessed Elekta Kaiku 27 times over the treatment period (range 7–86, median 23.5) [22]. Their monthly time spent in the ePRO platform was 49 min (mean), ranging from 6 to 233 min [22].

In the study period, nurses logged into the Elekta Kaiku platform a total of 393 times, with a mean of 14.6 logins per week (range 3–28) [22]. The physicians used the patient’s ePRO in the print-out and did not actively log into the platform.

At 6 months, 19 patients completed the Patient Feedback Form [21], as outlined in Table 3. The positive responses ranged from 52.6 to 70.6%, indicating that most patients strongly agreed that ePRO was simple to use and understand, improved communication with clinicians, and could be recommended to others.

**Table 3:** Evaluation of weekly ePRO utilizing the Patient Feedback Form (*n* = 19) [21]

Items	Too short	Just right	Too long
1. Time it took to complete		100%	
	**Not often enough**	**Just right**	**Too often**
2. Number of times completing	5.3%	89.5%	5.3%
	**Strongly agree**	**Agree**	Disagree*
3. Easy to complete	57.9%	36.8%	5.3%
4. Completing was useful	62.2%	31.6%	5.3%
5. Easy to understand	63.2%	26.3%	10.5%
6. Easier to remember symptoms and side effects	57.9%	26.3%	15.8%
7. Improved discussions with clinician	55.6%	27.8%	16.7%
8. Clinicians used information for my care	63.2%	26.3%	10.5%
9. The quality of care improved because of the questionnaire	61.1%	16.7%	22.2%
10. Communication with the clinician improved	52.6%	31.6%	15.8%
11. Made me more in control of care	57.9%	21.1%	21.1%
12. Recommend to others	63.2%	31.6%	5.3%
13. Would like to continue responding	70.6%	17.6%	11.8%

*Response category “strongly disagree” did not receive any responses (0%), so it has been omitted from the table

### Symptom burden

**Fig. 3:**
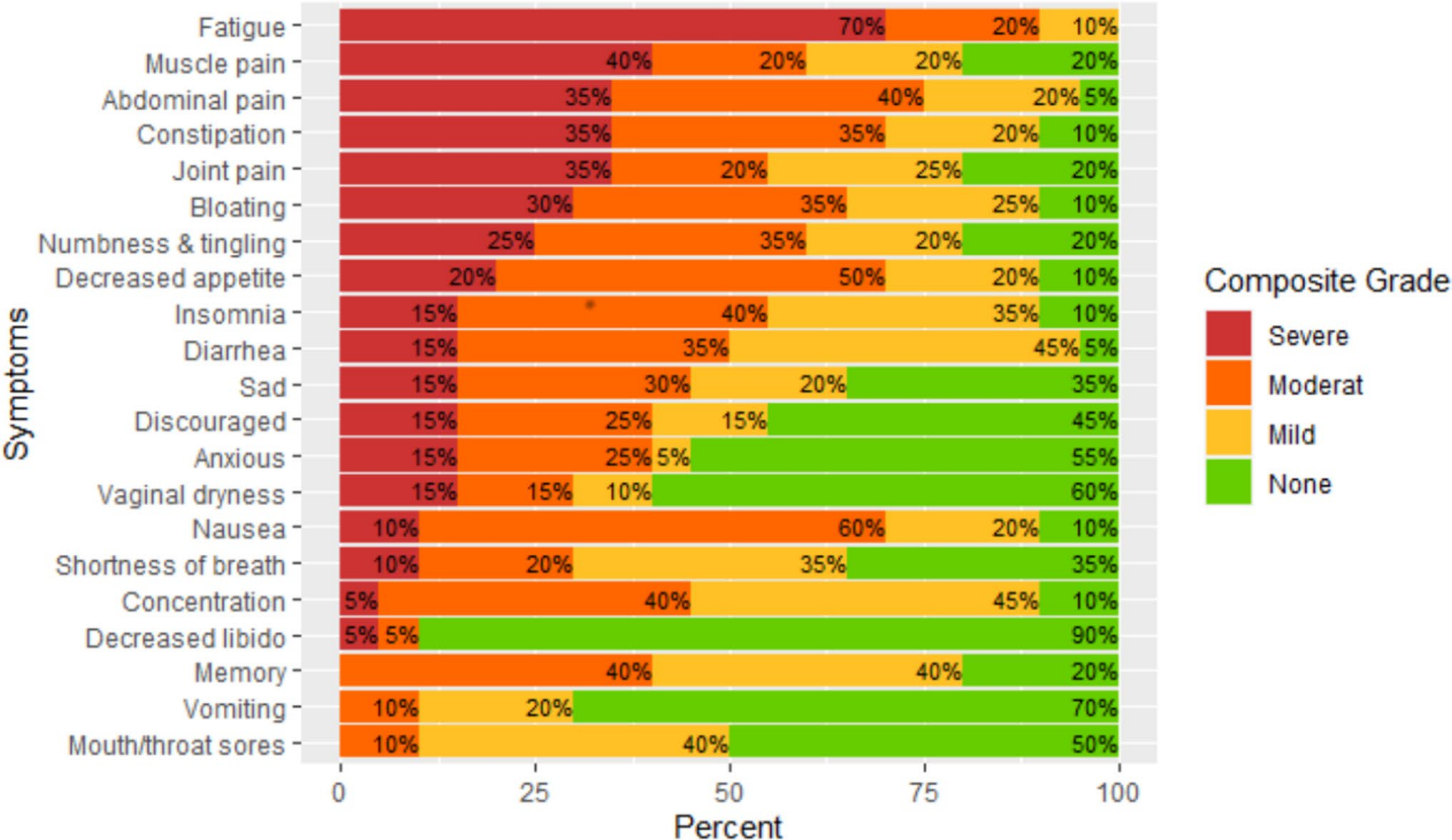
The symptom burden (PRO-CTCAE Composite Grade) across the entire treatment trajectory

During the intervention period, the eight most prevalent PRO-CTCAE symptoms (Grade 3) were fatigue (70%), muscle pain (40%), abdominal pain (35%), constipation (35%), joint pain (35%), bloating (30%), numbness and tingling (25%), and decreased appetite (20%) (Fig. 3) [29]. Three symptoms (memory, vomiting, and mouth/throat sores) were not reported at any time point as grade 3.

The patient advisory board was introduced to the Danish version of Elekta Kaiku prior to the study. They contributed with their perspectives on item selection and the content and modules in the ePRO platform and actively created peer-to-peer videos for the platform [16, 22]. The advisory board expressed admiration for the innovative design and various modules, firmly believing in its potential to benefit future patients.

## Discussion

To the best of our knowledge, this study is the first to investigate ePRO-based nurse-led consultations in women with ovarian and endometrial cancer undergoing platinum-based chemotherapy. Overall, the results indicate that the intervention may be feasible. A high ePRO completion (87%) was demonstrated during chemotherapy, with only 3 weeks with a response rate below 80%, and further 86% of the patients responded to ≥ 80% of weekly ePRO. This corresponds with the findings of Møller et al. [18] who found an 85% weekly ePRO adherence rate in patients receiving radiotherapy.

In the current study, patients received immediate feedback on their ePRO responses, and their responses were reviewed by nurses who followed the study-specific algorithm, which may have encouraged patients to continue reporting. These results align with other Danish ePRO studies [32], demonstrating a general interest and willingness for weekly ePRO reporting among cancer patients. Importantly, no safety issues were identified during this study, which is essential when transitioning responsibility from physician-led to nurse-led consultations and ensuring appropriate handling of ePRO. Skorsted et al. [33] conducted a qualitative evaluation of an eHealth lifestyle intervention and found that nurse-led consultations were perceived as a safe and appropriate alternative to traditional physician-led consultations and that patients expressed a high level of trust in the healthcare system. A future qualitative study related to our study, involving participating patients’ and healthcare professionals’ perspectives, will further investigate this.

The recruitment rate of 54% may be indicative of the predominantly older composition of this group and the date of recruitment, which occurred during a vulnerable phase when patients were initiating treatment for a potentially life-threatening cancer [34]. Further, our results are in line with findings from Kennedy et al. [14], who conducted an ePRO nurse-led study in the follow-up phase among an ovarian cancer population. Being the first study to test ePRO reporting during chemotherapy in the actual context, healthcare professionals may have been hesitant to enroll patients, a sentiment supported by studies reporting a variety of facilitators and barriers to implementing ePROs [6, 32].

The Elekta Kaiku ePRO platform is now operational in Denmark, and the patient advisory board has provided valuable user insights on the included modules, which enhances the applicability to real-life circumstances. The high patient satisfaction highlights the value and appreciation that patients place on the ePRO reporting. The practicability of this study is reasonable; only one patient experienced troubles with the ePRO platform, but this was due to an outdated device. Consequently, this study advanced without any further resources, leading to increased responsibility for the nurses and maybe also additional telephone contacts. In line with our results, Tolstrup et al. [35] identified a significantly higher number of telephone contacts in the ePRO intervention group, indicating an increased awareness of the symptoms.

As shown in Fig. 3, patients reported a high level of severe symptom burden (grade 3) throughout the treatment trajectory, with fatigue being the symptom with the highest severity (70%). This result is not surprising as fatigue is widely acknowledged as a significant symptom during chemotherapy. Beesley et al. [36] examined the frequency and trajectory of symptoms following first-line chemotherapy for ovarian cancer. Their results show that tiredness continues throughout and after treatment, highlighting the need for more research [36].

Furthermore, our data show a persistently significant symptom burden during chemotherapy, which underlines the need for interventions. Especially symptoms such as myalgia, arthralgia, and constipation may, to some degree, be preventable and treatable. Although abdominal pain was one of the symptoms included in the ePRO platform, patients added it to the free-text box [23, 29]. This could indicate the complexity of abdominal symptoms, subjectivity, and the difficulty of distinguishing between them, but it should be examined further. However, an important aspect to note is that numerous patients in the current study received hyperthermic intraperitoneal treatment as part of the surgical procedure, which may have resulted in an increased symptom burden [31]. Consequently, healthcare providers must adopt proactive approaches to reduce the symptom burden. It is documented that clinicians underestimate the patients’ symptom burden and our findings indicate inadequate clinical management [6]. This problem is prevalent not just in the field of gynecological cancer but also among other cancer populations, and the ePRO-based methodology described here may be useful in other contexts. Further investigation is required, and this current study adds to the preliminary body of evidence.

Additionally, we found that 55% of the planned nurse-led consultations were conducted by nurses. This result reflects the complex symptom burden experienced by the patients, necessitating physician involvement for prescribing medications to reduce immunological hypersensitivity and infections, adjusting chemotherapy doses in terms of peripheral neuropathy, and delivering genetic testing results, hindering nurse-led consultations. In accordance with local procedures, genetic testing should be delivered by a physician, a change that was implemented during the study. This was essential, as the genetic testing results influenced decisions on whether patients could receive maintenance therapy following chemotherapy, requiring the consultation of a physician [37]. Further, the results denote that specialized oncology nurses may be able to facilitate nurse-led consultations, but it did present certain challenges when attempting to integrate it into existing clinical prioritization and workflow. While this study was running, we experienced a nursing staff shortage, which might have resulted in insufficient resources for nurses to perform consultations, reducing the number of facilitated nurse-led consultations. Prior to initiating this study, appropriate knowledge and experience were considered to be important, and the nurses were provided with training and given a manual describing the study’s unique procedure. Despite this, some nurses may have lacked prior experience facilitating nurse-led consultations, perhaps contributing to reluctance or insecurity [9]. As indicated, multiple explanations exist for the 55% success rate of nurse-led consultations, but the results show first and foremost that this patient population has a significant symptom burden during chemotherapy, demonstrated for the first time through an ePRO platform in Denmark. We believe a 55% success rate is acceptable, but leaves room for improvement, and as a consequence, we have adjusted local workflows to improve this rate. Adapting new practices and electronic systems takes time, and adequate resources are required for successful implementation [38]. The inverted relationship between technology, workflow in the actual context, and personal characteristics may impact the implementation of nurse-led ePRO-based consultations [38].

Overall, the use of ePRO in this ovarian-endometrial population provides an evidence-based approach to assessing the patients’ symptom burden during oncological treatment, and it can provide essential information to clinicians and thus improve clinical symptom management [6]. The study’s findings are pivotal for understanding the benefits and challenges of ePRO reporting and for developing strategies for future utilization.

The small sample size and recruitment from a single site decrease the generalizability of this study. While the sample is diverse, representing women with ovarian or endometrial cancer, there is a risk of selection bias as the sample predominantly includes younger women with proficient technological abilities [39]. Lastly, the involvement of the patient advisory board is a strength in ensuring a patient-friendly ePRO platform with content that aligns with patients’ needs.

## Conclusions

The results of this study indicate that weekly ePRO reporting, combined with nurse-led consultations, may be feasible among women undergoing platinum-based chemotherapy for ovarian or endometrial cancer. The overall high completion and adherence rates, as well as the level of patient satisfaction, suggest the effectiveness of ePRO reporting in this population. Notably, nurse-led consultations were successful in 55% of the planned sessions, underscoring the complex and significant symptom burden experienced by patients and necessitating interventions from physicians. Given the challenges patients experience during chemotherapy, early interventions are essential to prevent symptom progression. This study emphasizes that ePRO reporting might be a rich source of real-life data that may benefit future patients. Lastly, this patient-centered ePRO-based model might be beneficial in other contexts and should be further explored in a larger-scale study, as the small sample size limits its generalizability.

## Supplementary Information

Below is the link to the electronic supplementary material.

Supplementary file 1 (DOCX 30.8 KB)

Supplementary file 2 (DOCX 125 KB)

## Data Availability

No datasets were generated or analysed during the current study.
