# Supplementary material for: Systematic nurse-led consultations based on electronic patient-reported outcomes for women with endometrial or ovarian cancer during chemotherapy—a feasibility study
Source: Support Care Cancer. 2025 Sep 17;33(10):858. doi: 10.1007/s00520-025-09875-y (PMC12443879; doi:10.1007/s00520-025-09875-y)
Supplement: Supplementary file 2 — (DOCX 125 KB) [file 520_2025_9875_MOESM2_ESM.docx]

**Supplementary File 2.**

Symptoms selected from the PRO-CTCAE library and included in the ePRO platform powered by Elekta Kaiku [1–4].

| **Symptoms included in the ePRO platform** |
| --- |
| Constipation |
| Diarrhea |
| Mouth/Throat sores |
| Nausea |
| Vomiting |
| Bloating |
| Fatigue |
| Abdominal pain |
| Decreased appetite |
| Joint pain |
| Muscle pain |
| Numbness & tingling |
| Anxious |
| Concentration |
| Discouraged |
| Insomnia |
| Memory |
| Sad |
| Decreased libido |
| Vaginal dryness |
| Shortness of breath |

[1] Dueck AC, Mendoza T, Mitchell SA, et al. Validity and Reliability of the U.S. National Cancer Institute’s Patient-Reported Outcomes Version of the Common Terminology Criteria for Adverse Events (PRO-CTCAE). *JAMA Oncol* 2015; 1: 1051–1059.

[2] Christiansen MG, Pappot H, Jensen PT, et al. A multi-method approach to selecting PRO-CTCAE symptoms for patient-reported outcome in women with endometrial or ovarian cancer undergoing chemotherapy. *J Patient Rep Outcomes* 2023; 7: 1–13.

[3] Elekta Kaiku, https://www.elekta.com/products/life-sciences/elekta-kaiku/ (accessed 20 March 2025).

[4] PRO-CTCAE. PRO-CTCAE® Measurement System website, https://healthcaredelivery.cancer.gov/pro-ctcae/.
